# Rosai-Dorfman disease affecting the nasal cavities and paranasal sinuses

**DOI:** 10.1590/0100-3984.2015.0167

**Published:** 2016

**Authors:** Bruno Niemeyer de Freitas Ribeiro, Edson Marchiori

**Affiliations:** 1 Instituto Estadual do Cérebro Paulo Niemeyer, Rio de Janeiro, RJ, Brazil.; 2 Universidade Federal do Rio de Janeiro (UFRJ), Rio de Janeiro, RJ, Brazil.

**Figure 1:**
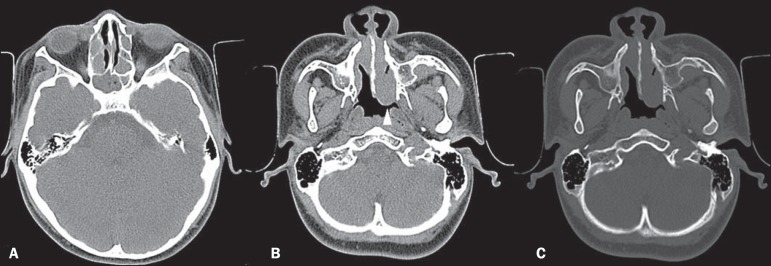
**A:** Axial CT section without contrast, showing opacification of the sphenoid sinuses and ethmoid cells by hypointense material. **B:** Axial CT section without contrast, showing a homogeneous, hypointense polypoid formation (arrowhead) in the left nasal cavity. **C:** Axial CT section, with a bone window setting, showing that there is no associated bone erosion.

*Dear Editor*,

Here, we report the case of a 17-year-old male who presented with a three-month history of nasal obstruction, asthenia, and febrile episodes. Physical examination revealed bilateral enlargement of cervical and axillary lymph nodes, all of which were painless on palpation. Laboratory tests showed mild leukocytosis, an elevated increased C-reactive protein level, and a high erythrocyte sedimentation rate. The venereal disease research laboratory test and monospot test were both negative, as was serology for HIV, toxoplasmosis, and cytomegalovirus. Computed tomography (CT) of the sinuses showed multiple, homogeneous, hypointense, rounded polypoid masses, which effectively narrowed the nasal passages, together with opacification of the ethmoid cells and sphenoid sinuses, with no evidence of bone erosion (Figure 1). Biopsies of a cervical lymph node and nasal lesions were negative for neoplasia and acid-fast bacilli, showing diffuse lymphoplasmacytic infiltration, foamy histiocytes, and emperipolesis. Immunohistochemistry showed positivity for S-100 protein, positivity for CD68, and negativity for CD1a. A diagnosis of Rosai-Dorfman disease was made, and corticosteroid therapy was started, resulting in slow, progressive improvement.

Recent studies in the radiology literature of Brazil have stressed the importance of CT and magnetic resonance imaging (MRI) in improving the diagnosis of head and neck masses^(1-5)^. Rosai-Dorfman disease, also known as sinus histiocytosis with massive lymphadenopathy, is a rare, benign lymphoproliferative, usually self-limiting, condition characterized by bilateral, painless cervical lymphadenopathy^(6-11)^, with spontaneous resolution in approximately half of all cases^(7)^. The disease has a slight predilection for males and primarily affects children, adolescents, and young adults, 80% of cases occurring in individuals under 20 years of age^(6)^. Extranodal involvement occurs in 30-40% of al cases^(6,8,9)^, being most common in immunocompromised individuals and preferentially affecting the skin, respiratory tract, reticuloendothelial system, genitourinary tract, or bones^(6,8,9)^. Although uncommon, enlargement of the mediastinal, hilar, axillary, and inguinal lymph nodes can occur.

The etiology of Rosai-Dorfman disease is unclear, although it could be related to changes in the immune response or to infections caused by agents such as varicella-zoster virus and other herpes viruses, as well as Epstein-Barr virus, cytomegalovirus, *Brucella* spp., and *Klebsiella* spp.^(6,7,9,11)^.

Imaging tests such as CT and MRI are useful for evaluating the extent of Rosai-Dorfman disease, although there are no specific characteristics. When it affects the paranasal sinuses, it typically manifests as polypoid masses, mucosal thickening, with or without bone erosion, with preferential involvement of the maxillary sinuses and ethmoid cells^(9,10)^. The diagnosis is established by histopathology^(8)^.

The differential diagnoses include several types of lymphoreticular malignancy, such as lymphoma, malignant histiocytosis, and monocytic leukemia, which have histopathological features similar to those of Rosai-Dorfman disease but present atypia and a rapid, aggressive evolution. Another major differential diagnosis is Kikuchi-Fujimoto disease (histiocytic necrotizing lymphadenitis), the clinical profile of which resembles that of Rosai-Dorfman disease, with cervical lymphadenopathy, although the former predominantly affects females and manifests as necrotizing histiocytosis on histopathology^(12,13)^.

In Rosai-Dorfman disease, the treatment modality of choice and the timing of treatment are controversial. Nevertheless, the choice of treatment strategies depends on the severity of the disease, mild cases being managed with observation only, whereas cases that are more severe are typically managed with corticosteroid therapy, chemotherapy, radiotherapy, or surgery^(6-11)^.

In conclusion, although Rosai-Dorfman disease does not present specific imaging characteristics, it should be considered among the diagnostic possibilities in cases of painless bilateral cervical lymphadenopathy, particularly in children and adolescents.
